# Salivary extracellular vesicles in precision periodontology: diagnostic biomarkers and therapeutic opportunities

**DOI:** 10.3389/fdmed.2026.1964471

**Published:** 2026-10-13

**Authors:** Amanchi Vineela, Amita Rao, Srilakshmi Mahadevan

**Affiliations:** Department of Periodontology, AB Shetty Memorial Institute of Dental Sciences (ABSMIDS), Nitte (Deemed to be University), Mangalore, India

**Keywords:** biomarkers, extracellular vesicles, periodontitis, precision periodontology, saliva

## Abstract

Periodontitis is a chronic inflammatory disease in which clinical and radiographic assessments reflect the consequences of tissue destruction rather than ongoing biological activity. Salivary Extracellular Vesicles (EVs) have emerged as a promising source of minimally invasive biomarkers capable of reflecting real-time periodontal biology, owing to their stable, membrane-protected cargo of proteins, lipids, and regulatory nucleic acids. Current studies suggest that EVs-associated microRNAs and proteins may help distinguish periodontal health from disease and may reflect biological changes following periodontal therapy. This mini review discusses the biological origin and cargo of salivary EVs and examines their emerging relevance to precision periodontology. EVs derived from mesenchymal and periodontal ligament stem cells also show immunomodulatory, osteogenic, and regenerative effects in experimental models. Although diagnostic and therapeutic evidence is still emerging, growing findings support the potential of EVs-based approaches in periodontology. Further progress through standardised EVs isolation and characterisation, robust biomarker validation, and well-designed clinical studies will facilitate their translation into personalised periodontal care.

## 1 Introduction

The Global Burden of Disease 2021 study estimated that periodontitis affected approximately 951 million people aged 15–69 years worldwide, with peak prevalence in individuals aged 50–59 years (1). Periodontitis is a chronic biofilm-associated inflammatory disease characterised by progressive destruction of the tooth-supporting tissues, resulting in periodontal pocket formation, Clinical attachment loss, and alveolar bone resorption (2). Although significant advances have been made in periodontal therapy, disease diagnosis and monitoring still rely predominantly on clinical and radiographic assessments, which primarily reflect past tissue destruction rather than ongoing biological activity (3). The retrospective nature of conventional diagnostics limits early detection of active disease and individualised assessment of treatment response, creating a clear need for minimally invasive biomarkers capable of reflecting real-time periodontal biology rather than its historical consequences.

Extracellular vesicles (EVs) are membrane-enclosed particles released by cells that mediate intercellular communication and regulate inflammation, immune responses, and tissue repair through the transfer of bioactive proteins, lipids, and nucleic acids (4–6). Among the biofluids used for EVs isolation, saliva is particularly attractive for periodontal applications because it can be collected non-invasively, enables repeated longitudinal sampling, and directly reflects the local oral environment, making it a candidate source of molecular information (7, 8).

Precision medicine aims to deliver personalised healthcare by integrating an individual's genetic, molecular, phenotypic, and environmental characteristics into clinical decision-making (9, 10). This paradigm has transformed several medical specialities, but its application in periodontology still remains limited by the lack of reliable biomarkers that accurately reflect disease activity and progression (3, 10, 11). In this context, salivary EVs have emerged as promising biomarkers owing to their stability and their ability to carry proteins, lipids, and nucleic acids that reflect the periodontal microenvironment (12–14). Beyond their diagnostic potential, EVs derived from regenerative cell sources, including mesenchymal and periodontal ligament stem cells (PDLSCs), carry bioactive molecules that modulate inflammation and tissue repair (15, 16).

This review summarises the biology of salivary EVs, evaluates their emerging role as diagnostic and disease monitoring biomarkers, examines their therapeutic applications in periodontal regeneration, and identifies the key translational challenges to their integration into precision periodontal care.

## 2 EVs biology

EVs are lipid bilayer-enclosed particles released by cells that lack the ability to replicate independently and encapsulate proteins, lipids, Deoxyribonucleic acid, messenger RNAs (mRNAs), and microRNAs (miRNAs) (7, 17). The phospholipid bilayer protects this cargo from enzymatic degradation, which enhances molecular stability, preserves biological activity, and underlies much of EVs diagnostic and therapeutic potential. The International Society for Extracellular Vesicles (ISEV), through its Minimal Information for Studies of Extracellular Vesicles (MISEV) guidelines, recommends using the generic term “EVs” rather than subtype-specific terms such as exosomes or ectosomes unless their specific biogenesis pathways and defining characteristics have been experimentally established. The concept and definition of exosomes have evolved over time, and current EVs terminology emphasises experimentally demonstrated biogenesis and molecular characteristics rather than size or physical properties alone. Because commonly used isolation methods do not allow definitive separation of pure exosomes from other EVs populations based solely on physical properties, the term “exosome” should therefore be used cautiously unless endosomal origin and relevant molecular characteristics have been experimentally demonstrated (17).

Based on their biogenesis and mechanism of release, EVs are broadly classified into exosomes, microvesicles (MIVs), apoptotic bodies, and other emerging subtypes, each differing in size, morphology, molecular cargo, and biological function (7, 18). The term “exosome” was originally introduced to describe membrane-bound vesicles enriched in 5′-nucleotidase activity (19). Exosomes arise through the endosomal pathway, in which inward budding of endosomal membranes forms multivesicular bodies that subsequently fuse with the plasma membrane (20, 21). MIVs(ectosomes), by contrast, form through outward budding of the plasma membrane involving cytoskeletal remodelling, and are released by several cell types including mesenchymal and adipose-derived stem cells (22, 23). Apoptotic bodies are larger vesicles formed during programmed cell death through membrane blebbing and may contain organelles, membrane fragments, and nuclear material that are subsequently cleared by phagocytic cells (22, 24).

Salivary EVs and their molecular cargo, comprising bioactive molecules carried within or associated with these vesicles, such as miRNAs, mRNAs, proteins, and cytokines, may influence local and systemic inflammatory processes (25).

### 2.1 Regulation of EVs biogenesis

EVs biogenesis and release are tightly regulated by intracellular membrane-trafficking machinery and are influenced by the physiological and pathological state of the parent cell (26–28). Exosome formation proceeds through both endosomal sorting complex required for transport (ESCRT) -dependent and ESCRT-independent pathways, whereas MIVs biogenesis is driven by cytoskeletal remodelling and outward plasma-membrane budding (26, 27). ESCRT-independent mechanisms can involve lipid-driven membrane remodelling, particularly ceramide generated through neutral sphingomyelinase activity, as well as tetraspanin-associated membrane organisation; these mechanisms may operate alongside Rab-family GTPases, calcium signalling, and cargo-sorting mechanisms that determine the molecular composition and downstream function of the released vesicles (27, 28).

In periodontitis, persistent microbial dysbiosis and inflammatory stimuli profoundly influence this regulatory machinery. Periodontal pathogens, particularly *Porphyromonas gingivalis,* along with associated inflammatory stimuli, increase EVs secretion while selectively modifying molecular cargo within the periodontal microenvironment (14, 15, 20, 29, 30). As a result, EVs released within the inflamed periodontal microenvironment become enriched with inflammation-associated proteins, nucleic acids, and regulatory RNAs that track the pathological status of the tissue (15, 30), providing the biological rationale for their investigation as salivary biomarkers in precision periodontology (15, 20, 31, 32).

### 2.2 Host versus bacterial origin of salivary EVs

Salivary EVs originate from both host cells and oral microorganisms (7, 31). Host-derived EVs arise from oral epithelial, immune, and glandular cells and carry proteins, lipids, nucleic acids, and cytokines that reflect host inflammatory and immune activity (7, 32). Outer membrane vesicles released by Gram-negative periodontal pathogens carry lipopolysaccharides (LPS), proteases, virulence factors, and bacterial nucleic acids (33, 34). During periodontitis, both populations participate in host–microbial communication and may jointly influence inflammation, immune activity, extracellular matrix degradation, and alveolar bone remodelling (33, 35). Their distinct molecular cargo therefore provides complementary information on host response and microbial activity within the periodontal microenvironment (32, 36).

### 2.3 Salivary EVs as diagnostic biomarkers

Salivary EVs have been proposed as non-invasive biomarkers for assessing periodontal disease activity and monitoring treatment response (15, 32). By reflecting changes in the periodontal environment, including pathogen-associated components, inflammatory and immune-related molecular signatures and clinical parameters associated with periodontal inflammation and tissue destruction and systemic status, these EVs may have potential applications in early disease detection, staging, and risk assessment (36, 37).

The diagnostic potential is largely attributed to disease-associated changes in molecular cargo, including miRNAs, mRNAs, proteins and EVs-associated markers (25, 36). In periodontitis, salivary EVs show increased levels of miRNA-140-5p, miRNA-146a-5p, miRNA-628-5p, PD-L1 mRNA, TNF-α mRNA, Interleukin-6 (IL-6), Interleukin-8 (IL-8), and global 5mC methylation, whereas miRNA-223-3p, OSX mRNA, Interleukin-10 (IL-10), CD9, and CD81 are decreased (12, 14, 38–42). EVs enrichment may further improve the detection of disease-associated miRNAs compared with unfractionated saliva. In a pilot cohort study, a combined salivary EVs-associated miRNAs panel discriminated periodontitis from periodontal health with an area under the curve of 0.96, supporting the diagnostic potential of salivary EVs-associated miRNAs. However, this was a pilot cohort and requires further validation in larger and more heterogeneous populations to establish the robustness and specificity of the biomarker panel, particularly in distinguishing periodontitis from other inflammatory conditions (12). Beyond diagnosis, EVs may provide dynamic biomarker information reflecting disease activity and response to therapeutic intervention. Alterations in EVs cargo following NSPT have been reported to parallel clinical improvement, suggesting potential applications in treatment-response monitoring, assessment of residual inflammation, patient stratification, and prognosis (32).

The biologic relevance of EVs-associated miRNAs is further supported by their regulatory roles in periodontal inflammation and bone metabolism. Microbial dysbiosis and LPS have been implicated in alterations in miRNAs expression and inflammatory signalling. Among these, miRNA-146a-5p, which is increased in salivary EVs in periodontitis, has been described as a negative-feedback regulator by targeting interleukin-1 receptor-associated kinase 1 (IRAK1) and tumour necrosis factor receptor-associated factor 6 (TRAF6), which may limit excessive NF-*κ*B activation, while miRNA-155-5p, upregulated following treatment in a non-EVs experimental periodontitis model, modulates the Th17/Treg balance (12, 43, 44).

Collectively, these EVs-associated miRNAs profiles constitute a molecular signature of inflammatory activity, immune regulation, and bone metabolism that underscores their potential as precision diagnostic biomarkers in periodontology, with proteins and other EVs-derived biomolecules offering complementary diagnostic information.

### 2.4 EVs-based therapeutic opportunities in periodontal regeneration

Beyond their diagnostic utility, EVs are increasingly investigated as cell-free therapeutic mediators for periodontal regeneration, owing to anti-inflammatory and immune-regulatory effects that make them potential cell-free approaches to periodontal regeneration (45). EVs released by mesenchymal stem cells (MSCs), PDLSCs, gingival MSCs, and other regenerative cell populations transport proteins, lipids, and regulatory RNAs capable of modifying recipient-cell behaviour; rather than replacing damaged tissue directly, they reproduce part of the paracrine signalling of their parent cells and may promote a microenvironment favourable for periodontal repair (15).

A major therapeutic property of EVs is their ability to regulate inflammation, which is a prerequisite for predictable healing given that persistent inflammation contributes to connective tissue destruction and impaired regeneration. MSCs-derived EVs have demonstrated immunomodulatory effects on macrophages, including modulation of inflammatory cytokine production. These effects may promote a shift toward a reparative macrophage phenotype, thereby reducing excessive or persistent inflammation and creating a more favourable microenvironment for subsequent tissue regeneration and new bone formation (15, 45–47).

EVs also exert direct regenerative effects on periodontal and bone-forming cells. Stem-cell-derived EVs enhance proliferation, migration, and osteogenic differentiation of PDLSCs and MSCs and promote mineralised tissue formation, acting on pathways associated with osteogenesis and bone remodelling (48). Preclinical studies suggest that pro-angiogenic EVs-derived signals may promote endothelial-cell migration and angiogenesis, potentially contributing to periodontal tissue regeneration (23, 49–51).

An emerging approach is the incorporation of EVs into hydrogels, scaffolds, and other biomaterial-based delivery systems to improve their retention and local availability at periodontal defects. These systems may facilitate controlled and sustained EVs release; however, their optimal formulation and therapeutic efficacy remain to be established (50, 52, 53).

Early human evidence is emerging. In a pilot proof-of-concept study by Puletic et al.,2024 reported improved clinical and inflammatory outcomes were reported following EV therapy in seven patients with Stage I–III periodontitis, although the small pilot sample limits efficacy conclusions (54). A retrospective case series by Froum et al., 2026 reported clinical improvements at 6 months in 13 patients with 23 periodontal defects treated with an exosome-containing regenerative protocol; however, the lack of a comparator and combined use of regenerative materials preclude determining the independent effect of exosomes (55).

Although encouraging, EVs evidence remains limited and is predominantly preclinical, with early human studies providing preliminary clinical observations. Standardisation of EVs isolation, characterisation, dosage, delivery, and potency assessment, together with adequately powered controlled studies and longer follow-up, is required to establish their clinical applicability.

### 2.5 Clinical implications in periodontology

The clinical relevance of EVs extends beyond their individual diagnostic and regenerative applications to their potential integration into periodontal decision-making (15, 25). Periodontitis is biologically heterogeneous, and patients with comparable clinical presentations may differ in inflammatory activity, host response, and microbial composition (15, 56, 57). Incorporating EVs-derived molecular information alongside conventional periodontal parameters could therefore provide a more comprehensive biological profile of individual patients and support increasingly individualised management strategies (15, 25).

The clinical interpretation of salivary EVs is complicated by their heterogeneous origins, which may include oral host cells, salivary glands, microorganisms, and potentially systemic sources. Consequently, establishing periodontal-specific EVs signatures remains an important challenge (7). Selective enrichment of host-derived EVs populations may improve detection of disease-associated molecular signatures; however, the reproducibility and clinical utility of such approaches require further validation. Altered levels of inflammatory mediators, including IL-6, IL-8, and IL-10, have been reported in enriched salivary EVs from individuals with periodontitis (14).

Changes in host EV subpopulations and associated cytokine cargo following NSPT have been reported, including differences between responders and suboptimal responders. A suboptimal treatment response was defined as the presence of >4 sites with PPD ≥5 mm at the clinical endpoint, whereas responders had ≤4 sites with PPD ≥5 mm. However, further longitudinal studies are required to determine whether these EV-associated changes reliably reflect disease activity or treatment response (32).

An exploratory longitudinal study by Wang et al., 2026 evaluated salivary host EVs subpopulations and cytokine profiles in 25 patients with Stage III–IV periodontitis before and 3 and 6 months after non-surgical periodontal therapy (NSPT). Changes in EVs subpopulations and cytokine cargo accompanied clinical improvement, with differences observed between responders and suboptimal responders. However, the exploratory design limits conclusions regarding their utility for monitoring treatment response (32).

EVs-based molecular profiles may provide additional information on biological activity and treatment response; however, their ability to distinguish active from stable disease or guide treatment selection has not yet been established. Clinical translation will require validated reference ranges, reproducible analytical workflows, and prospective evidence demonstrating added value over established periodontal assessment. Key studies investigating salivary EVs-associated molecular signatures and their potential applications in precision periodontology are summarised in Table 1.

**Table 1:** Salivary extracellular vesicle-associated molecular signatures and their potential applications in precision periodontology.

Author, year	Study design	EVs source	EVs biomarker/ cargo	Main finding	Potential Precision-periodontology application
Palanisamy V et al., 2010 (58)	Characterization study	Saliva	mRNAs	Demonstrated that human salivary exosomes contain a distinct and relatively stable mRNA cargo, supporting saliva-derived exosomes as a source of molecular information	Foundational evidence for salivary EVs-based molecular profiling
Ogawa Y et al., 2011 (59)	Proteomic characterization	Saliva	Proteomic analysis of two types of exosomes in human whole saliva	Identified two distinct salivary exosome subpopulations differing in size and protein cargo	Foundational salivary EVs characterization and phenotyping
Yu J et al., 2019 (39)	Case–control	Saliva	PD-L1 RNA	Reported altered salivary exosomal PD-L1 RNA in patients with periodontitis, suggesting potential association with periodontal disease status	Disease detection and severity/stage assessment
Nik Mohamed Kamal et al., 2020 (60)	Case–control profiling	Saliva and plasma	Exosome miRNAs signatures	Identified distinct circulating and salivary exosomal miRNAs profiles associated with chronic periodontitis	Molecular disease profiling and candidate biomarker discovery
Han P et al., 2021 (41)	Pilot clinical study	Saliva	Salivary EVs DNA methylation and bacterial OMVs associated Lipopolysaccharides	Differences in global DNA methylation and bacterial OMVs-related signals were observed across periodontal conditions, supporting their potential for periodontal-status discrimination	Disease discrimination and multimodal biomarker development
Han P et al., 2026 (61)	Exploratory study	Salivary microvesicles	Host microvesicles, host DNA methylome and microbial/OMVs profiles	Integrated host methylome and microbiome features of salivary microvesicles showed potential to distinguish periodontal health, gingivitis and advanced periodontitis	Molecular phenotyping disease discrimination and potential patient stratification

EVs, Extracellular Vesicles; mRNA, messenger RNAs; miRNAs, micro RNAs; RNA, Ribonucleic Acid; PD-L1 RNA, Programmed Death-Ligand 1 RNA; DNA, Deoxyribonucleic acid; OMVs, Outer Membrane Vesicles.

## 3 Conclusion

Salivary EVs represent a potential source of molecular information that may complement conventional periodontal assessment. Current diagnostic evidence remains largely exploratory, while therapeutic applications of stem-cell-derived EVs are supported predominantly by preclinical studies with limited early human evidence.

## 4 Future research and gaps

Methodological heterogeneity in saliva collection, EVs isolation, characterisation, and molecular analysis remains a major barrier to translation. Standardised workflows are needed to improve reproducibility (17). Future studies should validate multimolecular EVs signatures in larger, diverse cohorts and establish their periodontal specificity and clinical utility. For therapeutic applications, controlled clinical studies are required to define optimal EVs source, dose, delivery, safety, and long-term efficacy.
